# Has COVID-19 Affected Cancer Screening Programs? A Systematic Review

**DOI:** 10.3389/fonc.2021.675038

**Published:** 2021-05-17

**Authors:** Ibrahim Alkatout, Matthias Biebl, Zohre Momenimovahed, Edward Giovannucci, Fatemeh Hadavandsiri, Hamid Salehiniya, Leila Allahqoli

**Affiliations:** ^1^ Kiel School of Gynaecological Endoscopy, University Hospitals Schleswig-Holstein, Kiel, Germany; ^2^ Department of Surgery, Charité–Universitätsmedizin Berlin, Berlin, Germany; ^3^ Department of Midwifery and Reproductive Health, Faculty of Nursing and Midwifery, Qom University of Medical Sciences, Qom, Iran; ^4^ Departments of Nutrition and Epidemiology, Harvard T. H. Chan School of Public Health, Boston, MA, United States; ^5^ Channing Division of Network Medicine, Department of Medicine, Brigham and Women’s Hospital and Harvard Medical School, Boston, MA, United States; ^6^ Cancer Research Center, Shahid Beheshti University of Medical Sciences, Tehran, Iran; ^7^ Social Determinants of Health Research Center, Birjand University of Medical Sciences, Birjand, Iran; ^8^ School of Public Health, Iran University of Medical Sciences (IUMS), Tehran, Iran

**Keywords:** cancer, screening, coronavirus disease 2019 (COVID-19), health care, diagnosis

## Abstract

**Background:**

Health care services across the world have been enormously affected by the onset of the coronavirus disease 2019 (COVID-19). Services in oncology have been curtailed because medical services have been focused on preventing the spread of the virus and maximizing the number of available hospital beds. The present study was designed to investigate the impact of COVID-19 on cancer screening.

**Methods:**

Databases such as Medline, Web of Science Core Collection (Indexes = SCI-EXPANDED, SSCI, A & HCI Timespan) and Scopus were searched comprehensively for articles published until January 2021. The keywords used were COVID-19 and *cancer screening*, Articles dealing with cancer screening in the COVID-19 pandemic were included in the review.

**Results:**

The review comprised 17 publications. The impact of COVID-19 was categorized into four dimensions: a significant decline in cancer screening and pathology samples, the cancer diagnosis rate, an increase in advanced cancers, mortality rate and years of life lost (YLLs).

**Conclusion:**

Cancer screening programs have been clearly interrupted since the onset of the COVID-19 disease. The anticipated outcomes include delayed diagnosis and marked increases in the numbers of avoidable cancer deaths. Urgent policy interventions are needed to handle the backlog of routine diagnostic services and minimize the harmful effects of the COVID-19 pandemic on cancer patients.

## Background

Coronavirus disease 2019 (COVID-19), also known as severe acute respiratory syndrome coronavirus 2 (SARS-CoV-2), which began in December 2019, has now reached every corner of the world (1). COVID-19 has affected 221 countries. More than 100 million cases of the disease were registered until 27 January 2021, which makes it the worst pandemic in modern history (2). Health care services across the globe have been profoundly affected by COVID-19, and the consequences of the pandemic are yet unforeseeable (3, 4). A large number of medical procedures, elective and non-urgent scheduled surgeries, and elective visits have been canceled or rescheduled (5, 6). Telehealth is used for non-acute issues in some countries (7), while population-based cancer screening programs have been suspended in others (5). The COVID 19 pandemic has had unprecedented effects on cancer screening and preventive care (8).

The enforcement of stay-at-home guidelines for reducing the risk of transmission (9) has influenced primary, secondary and tertiary preventive programs (7). Individuals with potential non-specific symptoms of cancer have faced obstacles in consulting a specialist (7), largely due to fear and anxiety about being infected by COVID-19 in a health care setting. Both, patients and personnel at hospital sites experience fear and anxiety (5). There has been a marked decline in cancer screening and diagnosis, the development of tumor markers, the use of imaging procedures, biopsies, colonoscopy, gastroscopy, sigmoidoscopy, stool tests, low-dose computed tomography (LDCT), mammography, Pap tests, human papillomavirus (HPV) testing, colposcopy, laparoscopy, and melanoma screening (6, 8, 10–22).

Cancers are known to develop long after exposure to carcinogens, such as tobacco or the human papillomavirus (HPV) (23) Thus, the carcinogenic process provides ample opportunity to detect early precancerous lesions and start interventions that reverse or delay the progression of disease (24). Delaying the initial quest for symptoms results in subsequent disease (7) and its irreversible consequences (17, 25, 26). Any interruption of secondary preventive programs delays the diagnosis and treatment of cancer in addition to facilitating advanced disease, increasing mortality rates and total years of life lost (YLLs) (8, 16, 17, 25–28).

The suspension of cancer screening and early detection was deemed necessary in the initial phase of the COVID-19 pandemic, as demonstrated by a marked interruption in cancer screening rates during this time. COVID-19 could not be controlled within the initially anticipated period of time. As a result, routine cancer screening and treatment have not yet returned to pre-pandemic levels.

We have a limited body of data concerning the impact of COVID-19 on cancer screening programs. The impact of the pandemic on oncology is unpredictable because of changes in the behavior of the virus and lack of knowledge about the disease. In the present comprehensive systematic review, we reviewed published reports to determine the impact of COVID-19 on cancer screening programs. Papers on cancer screening published during the pandemic were reviewed in regard of cancer diagnosis and its impact on mortality rates and YLLs. We provide recommendations for future actions that will mitigate the potential negative effects of anti-COVID-19 measures on cancer screening. Strategies to handle similar events on a global basis are also addressed.

## Methods

### Search Strategy and Information Sources

This systematic review was conducted in accordance with the systematic review checklist (PRISMA). The three databases PubMed/MEDLINE, Scopus, and Web of Science were searched for relevant articles. The search was performed on December 28, 2020 using the following keywords: “Early Detection,” “Cancer,” “Cancer Screening,” “Cancer Screening Tests,” “Early Diagnosis,” “COVID 19,” “Coronavirus Disease-19,” “Coronavirus Disease,” “SARS-CoV-2 Infection,” “SARS-CoV-2,” “2019-nCoV,” “Coronavirus, 2019 Novel,” “SARS CoV 2 Virus,” “Severe Acute Respiratory Syndrome Coronavirus 2,” “COVID-19,” “Coronavirus Disease 19,” “SARS Coronavirus 2”. Boolean (AND, OR) operators and the MeSH terms were used to optimize the selection of records.

### Inclusion and Exclusion Criteria

All types of observational studies conducted throughout the world, published exclusively in the English language, addressing cancer screening and diagnosis, cancer care, detection programs for early and appropriate detection and treatment were included in the review. Studies that did not address the specific effects of the coronavirus on cancer screening, included patients diagnosed with cancer prior to the pandemic, or patients with the symptomatic diagnosis of primary cancer, were excluded. Reviews, case reports, letters to editors, commentaries, and reports were also omitted. Studies were selected first by title and then by abstract. Their eligibility was then confirmed by a review of the full text. The PRISMA flow chart illustrates the process of selection.

### Screening of Studies and Study Selection

After removing duplicates, the studies were first selected according to the relevance of their titles and abstract (LA and HS). Next, full-text articles were reviewed to confirm eligibility (LA and IA). Articles that addressed any single aspect of cancer screening during the COVID-19 pandemic period were included. All retrieved articles were entered into a database on Endnote X7.

### Data Extraction and Data Items

We used a pre-prepared checklist for data extraction, the following information was extracted from each study: the first author’s last name, country, study design (source of the data), type of cancer and screening, study objectives, main findings, and recommendation.

### Quality Assessment

To assess the quality of articles included in the review, the Newcastle-Ottawa Quality Assessment Form was used. This tool contains of 3 separate sections: selection, comparison, and result. Studies scored based on the overall scores and divided into 3 categories: Good, Fair and Poor (29).

## Results

### Selection of Studies

**Figure 1:**
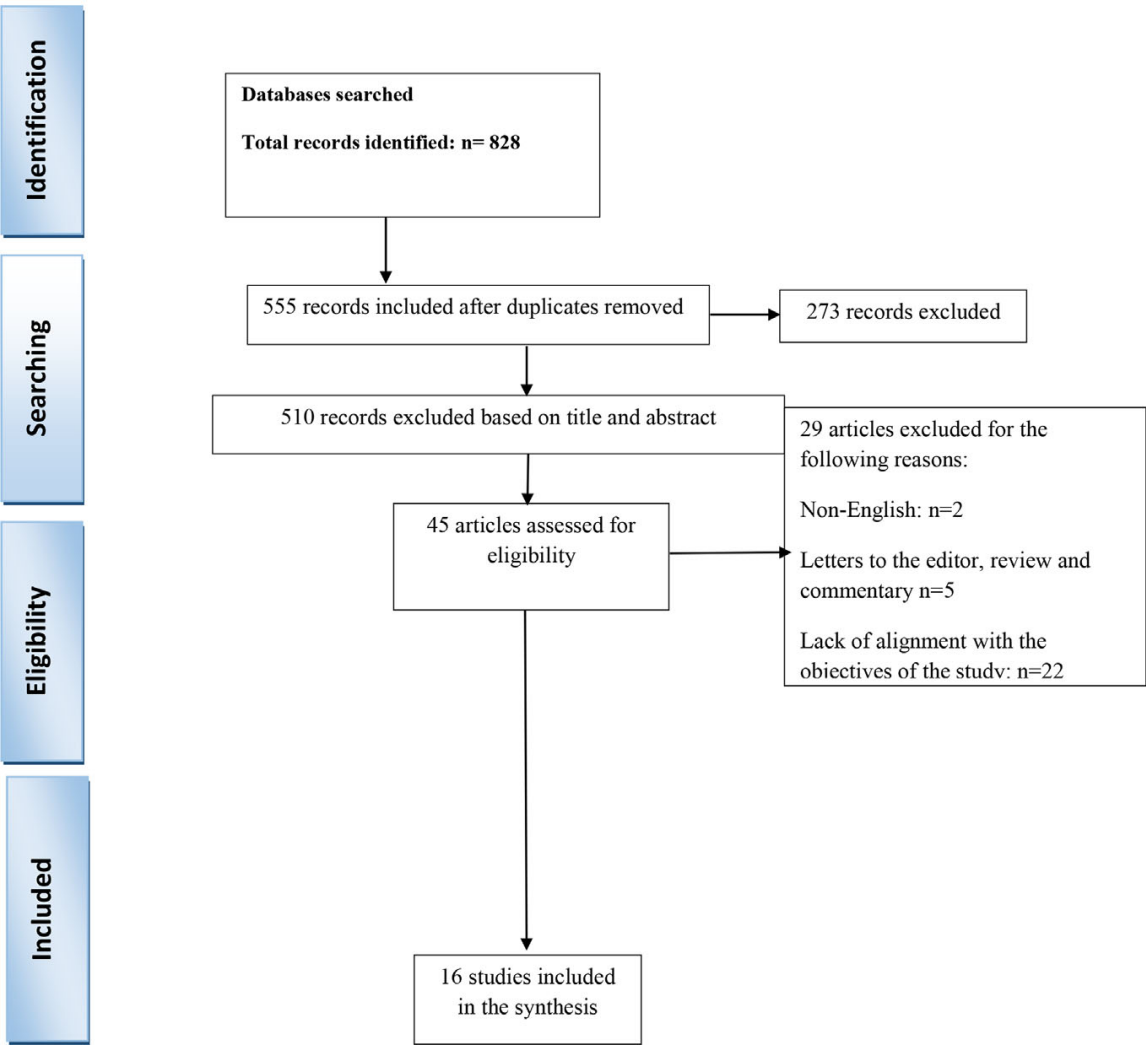
Study flow diagram.

The various databases yielded a total of 828 publications, of which 273 were duplicate articles. Of the remaining 555, 510 were omitted after reviewing the title and abstract. Seven of the remaining 45 articles were omitted for the following reasons: two were non-English publications and five were letters to the editor, review or commentaries. After a review of full texts, a further 22 articles were omitted because the contents were not aligned to the objectives. Finally, 16 studies were included in a systematic review (**Figure 1**).

### Study Characteristics

Seventeen studies deemed eligible for the investigation were divided into the following four categories: cancer screening and pathology samples (3, 8, 14, 16, 17, 27, 28, 30–33), cancer diagnosis in the COVID-19 period and factors related to the reduced diagnosis of cancer (8, 17, 26, 27, 31, 34, 35), impact of lockdown-related delay of medical care on tumor stage at the time of diagnosis (17, 25, 27, 30, 32–34, 36–38), mortality rate and YLLs (17, 38). Based on our review using the relevant checklist, 15 studies had good quality and 1 article had moderate quality.

#### Cancer Screening and Pathology Samples

COVID-19 has interrupted cancer screening such that, during the pandemic, reduced rates of cancer were reported from all screening programs (3, 8, 14, 16, 17, 27, 28, 30–33). The effect was more pronounced in countries with a greater prevalence of COVID-19 or poorly controlled rates of COVID-19 infection. Compared to the pre-COVID period, colonoscopy rates fell by 4.1% to 75% (8, 28). Gastroscopies, prostate, and lung screening rates were reduced by 57%, 74%, and 56%, respectively (28, 30). Screening mammograms declined by 22.2-85% (3, 16, 28, 32). We attribute this difference to the prevalence of COVID-19 in various countries.

Cancer screening rates have dropped at all levels of hospital care and in nearly all age groups (38).

Furthermore, the histopathological investigation of cancer screening-related samples has been severely affected by COVID-19. The histopathological and cytological workload was reduced by 35-72% compared to the preceding three years (14). Reductions in cancer biopsies were reported for breast (−31 to -71%), colon (-33 to -79%), and lung cancer (-47 to -58%) (28). In Belgium, de Pelsemaeker and registered a significant drop in the number of breast resection specimens and pulmonary endobronchial ultrasound (EBUS) biopsies (14). The impact of COVID-19 on cancer screening and pathology samples is summarized in **Table 1**.

**Table 1:** Impact of COVID-19 on cancer screening and pathology samples.

First Author	Country	Study Design/source of the data	Quality Assessment	Screening type	Main result
**Cheng** (8)	Taiwan	Prospective observational study (University Hospital screening)	Good	Colonoscopy (fecal immunochemical test)	Screening uptake rates were 88.8% between December 2019 and April 2020, and 91.2-92.7% in the preceding three years. The colonoscopy rate during the pandemic (66.1%) was significantly lower than that in the preceding three years (70.2–77.5%). Up to 10.9% of screening investigations were rescheduled or canceled, which was significantly higher than the cancellations in the preceding three 3 years. A half of the cancellations were due to fear of infection.
**De Pelsemaeker** (14)	Belgium	Prospective observational study (Cliniques universitaires Saint-Luc)	Fair	Cancer screening (histological and cytological samples, immunohistochemistry and molecular tests)	The histological and cytological workup of colon biopsies, breast biopsies, and cervical cytology were reduced by fear of COVID-19 infection.
**Lang** (30)	USA	Retrospective review (LCS institution)	Good	LDCT	Annual and baseline LDCT volumes were reduced by approximately 72% and 78%, respectively. Follow-up LCS LDCT volume fell by approximately 50%.
**Lantinga** (31)	Netherlands	Retrospectively analysis (database registry from 20 Dutch hospitals)	Good	Gastrointestinal endoscopy	19,296 patients underwent endoscopy in 2019, and 9776 during the lockdown in 2020. Gastroscopies and colonoscopies fell by 57% (from 7846 to 4467) and 45% (from 12219 to 5609), respectively.
**Mizuno** (27)	Japan	Retrospective cohort study(tertiary emergency hospital)	Good	CRC	A drop in ambulant colonoscopy rates and emergency admissions at the start of the pandemic.
**Patt** (28)	USA	Retrospective analysis (clearinghouse database representing 5%-7% of the Medicare fee-for-service population)	Good	Cancer care (breast, colon, lung biopsy)	Screenings for breast, colon, prostate, and lung cancers were reduced by 85%, 75%, 74%, and 56%, respectively. Reduced biopsy rates were also observed in April and July for breast (−71% and −31%), colon (−79% and −33%), and lung (−58% and −47%) cancer E&M visits (−74%), new patient E&M visits (−70%), and established patient E&M visits (−60%) were significantly reduced.
**Song** (32)	USA	Retrospective analysis(data from a private health insurer, Independence Blue Cross (Independence)	Good	Breast cancer screening	The numbers of screening and diagnostic mammograms fell by 58% and 38%, respectively. According to estimations, it would take a minimum of 22 weeks to clear the queue of missed mammograms.
**Tsai** (3)	Taiwan	Retrospective analysis (A national screening database)	Good	Breast cancer	The total number of newly diagnosed cancers fell by 22.2% during the lockdown period.
**Van Haren** (33)	USA	Prospective observational study (institutional LDCT screening database)	Good	LDCT	Total monthly LDCT and monthly LDCT for new patients was significantly reduced during the COVID-19 pandemic compared to pre-pandemic levels (39 ± 40 *vs*. 146 ± 31), (15 ± 17 *vs*. 56 ± 14) The number of new patients decreased and the “no-show” rate was significantly increased from baseline (25%).
**Yin** (16)	USA	Retrospectively analysis (55 breast imaging centers from 27 states)	Good	Breast surgery, breast imaging	Breast surgery (20.5%), breast imaging (61.7%), and genetic consultations dropped to 39.9% of the average weekly volumes before COVID-19.
**Yong** (17)	Canada	Simulation modeling analysis (Canadian Cancer Registry)	Good	Cancer screening (breast and CRC)	A three-month interruption of breast cancer screening due to COVID-19 resulted in 644,000 fewer screens.

LDCT, Low-dose computed tomography (for lung cancer screening); LCS, Lung cancer screening; CRC, Colorectal cancer screening; E&M, hospital out-patient evaluation and management.

#### Cancer Diagnosis in the COVID-19 Period and Factors Related to the Reduced Diagnosis of Cancer

One of the consequences of reduced cancer screenings is a decline in cancer diagnosis rates (8, 17, 26, 27, 31, 34, 35). According to De Vincentiis et al., cancer diagnosis at the Pathologic Anatomy Unit serving a Secondary Care Hospital Network in the Province of Macerata, Italy during the COVID pandemic fell by 11% compared to the average numbers in the last few years (39% *vs* 50%). However, various reduction rates were noted for the different types of cancer (34) (**Table 2**). A study in the Netherlands reported a steep decline in the absolute number of “suspicious of gastrointestinal (GI) cancers” and “colon cancers” (31). In Canada, a three- and six-month interruption in the breast screening program caused a 7% and 14% drop in cancer diagnosis (17). A marked drop in newly diagnosed gynecological tumors (-24% to -49%) was reported in Austria during the COVID-19 pandemic, and the median age of the patients was significantly lower than that of patients diagnosed with cancer in 2019 (59.4 *vs*. 61.3 years). A nearly 10% decline was noted in the diagnosis of breast cancer (35). The impact of COVID-19 on cancer diagnosis is summarized in **Table 2**.

**Table 2:** Impact of COVID-19 on cancer diagnosis.

First Author	Country	Study Design/source of the data	Quality Assessment	Screening type	Main result
**De Vincentiis** (34)	Italy	Retrospectively analysis (Hospital based)	Good	Cancer diagnosis (cellular pathology)	Cancers were diagnosed in 50% during 2018 and 2019 compared to 39% in 2020. Reductions were most notable for prostate (75%), bladder (66%) and colorectal cancers (62%), moderate for breast cancer (26%), slight for gastric cancer (10%), minimal for lung cancer (2%), and no reductions were noted for metastatic and pancreatic cancers, and skin melanoma.
**Gathani** (26)	England	Retrospectively analysis (National database)	Good	Breast cancer diagnoses	Breast cancer diagnosis in the first half of 2020 was 28% lower than that during the same period in 2019.
**Lantinga** (31)	Netherlands	Retrospectively analysis (database registry from 20 Dutch hospitals)	Good	Gastrointestinal endoscopy	Detection of cancer decreased by 35.12% (from 524 to 340); the likelihood of detecting cancer during endoscopy increased (2.7% [95% confidence interval (CI) 2.5–3.0] in 2019 *vs*. 3.5% [95% CI 3.1–3.9] in 2020).
**Mizuno** (27)	Japan	Retrospective cohort study(tertiary emergency hospital)	Good	CRC	A drop in colorectal cancers detected by cancer screening.
**Tsibulak (** 35)	Austria	Retrospectively analysis (18 gynecological or breast cancer centers)	Good	Gynecological and breast cancer screening	A marked decline in newly diagnosed tumors since the lockdown (-24% in March 2020 versus March 2019, and -49% in April 2020 versus April 2019).
**Yong** (17)	Canada	Simulation modeling analysis (Canadian Cancer Registry)	Good	Cancer screening (breast and CRC)	A three- and six-month interruption would cause a 7% (from 28,500 to 26,600) and 14% (from 28,500 to 24,400) drop in diagnoses, respectively. This would be accompanied by an increase in non-screening-detected cancers of 550 and 1020 (10% and 19% increase) that year for three- and six-month interruptions, respectively, with the increase persisting for at least a year after screening resumes. A six-month interruption in colorectal screening would cause the early diagnosis of 19,000 adenomas and colorectal cancers to be missed.

CI, Confidence interval; CRC, Colorectal cancer screening.

Fear of being infected by COVID-19 on the part of patients (8), halted screening programs in hospitals, the re-deployment of staff towards critical care for the management of patients with COVID-19, and changing strategies in hospitals for the prevention of nosocomial infection (27) were reasons for fewer cancers being diagnosed during the COVID-19 pandemic.

#### Impact of Lockdown-Related Delay of Medical Care on Tumor Stage at the Time of Diagnosis

**Figure 2:**
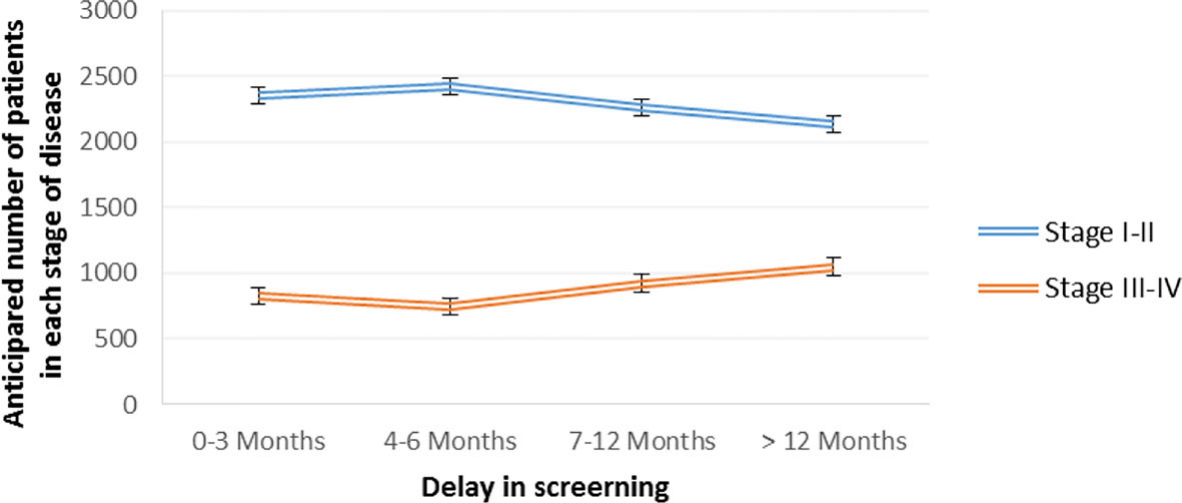
Estimated colorectal cancer progression due to delayed diagnosis in various intervals, based on Ricciardiello et al. (25).

The interruption of cancer prevention due to suspended cancer screenings may delay the diagnosis (17, 25, 33, 34, 36, 37), increase the numbers of symptomatic patients (27), and disclose cancers in more advanced stages (36). In an investigation of 3184 patients, Ricciardiello et al. concluded that a delay of 7-12 and > 12 months would lead to a significant 3% and 7% increase in the detection of advanced cancers (25) (**Figure 2**).

A study in Canada showed that three- and six-month interruptions in breast and colorectal cancer screening would lead to more numerous cases being detected in a late stage (stage IIIA or worse) between 2020 and 2029. Besides, longer interruptions (12 months) would have a greater impact on the spike in advanced cancers (17). The impact of lockdown-related delays in medical care on tumor stage at the time of diagnosis is summarized in **Table 3**.

**Table 3:** Impact of lockdown-related delays in medical care on tumor stage at the time of diagnosis.

First Author	Country	Study Design/source of the data	Quality Assessment	Screening type	Main result
**D’Ovidio** (37)	Italy	Retrospective controlled cohort study (Hospital base)	Good	CRC	The “high-risk” adenomas detection rate was significantly higher in the “lockdown group” than in controls (47% *vs*. 25%). Colorectal cancers were more numerous in the “lockdown group” than in controls (8% *vs*. 1%). Multiple regression analysis showed that the lockdown period (HR, 2.2) was an independent predictor of high-risk adenomas and colorectal cancers
**Van Haren** (33)	USA	Prospective observational study (institutional LDCT screening database)	Good	LDCT screening	Lung nodules suspicious for malignancy (Lung-RADS 4) after resumption of routine surgery were increased by 21%. Enlarged nodules after surgery were increased by 31.3%.
**Yong** (17)	Canada	Simulation modeling analysis (Canadian Cancer Registry)	Good	Cancer screening, (breast and CRC)	A three- and six-month service interruption would lead to 310 and 670 more cancers detected in an advanced stage (stage IIIA or higher). A 12-month interruption of breast cancer screening will be followed by 62% of cancers in advanced stages. A three-month or six-month screening interruption would lead to 1100 or 2200 more colorectal cancers, respectively; and more than 60% of cases would be in an advanced stage (III or IV).

CRC, Colorectal cancer screening; HR, hazard ratio; LCS LDCT, lung cancer screening low-dose computed tomography scan; Lung RADS, Lung CT Screening Reporting and Data System.

According to predictions, once the lockdown has been lifted it will take a minimum of 12-24 weeks to clear the queue of missed cancer screenings (32, 38). Lang and coworkers reported that, after resuming screening, a mere 68% of the Lung Cancer Screening (LCS) reached the average weekly pre-COVID volume in the following 10 weeks (30). According to Maringe et al., even after all restrictions on cancer screening have been lifted, it will take 3-6 months for screening figures to return to pre-pandemic levels (38).

#### Mortality Rate and YLLs

The effect of delayed cancer diagnosis will not be perceived in the immediate future alone; premature deaths may occur as long as five years later (17, 38). According to a study performed in Canada, cumulative excess deaths following the interruption of cancer services will continue to rise well beyond 2029 (17).

In a study conducted in England, death rates were compared with pre-pandemic figures. Increased death rates are anticipated up to five years after the diagnosis. Mortalities secondary to breast cancer (total number of breast cancer n=32,583) will increase annually from 965 (95% CI 958–972) to 1028 (1019–1036). Annual deaths due to lung cancer (total number of lung cancer n=29,305) are estimated to increase from 18,443 (95% CI 18,388–18,503) to 19,855 (19,804–19,901). Deaths due to colorectal cancer (total number of colorectal cancer n=24,975) will increase annually from 5051 (95% CI 5004–5099) to 6078 (6032–6140). Corresponding figures for esophageal cancer (n=6744) will be 3656 (95% CI 3642–3670) to 4034 (4017–4050) (38).

**Figure 3:**
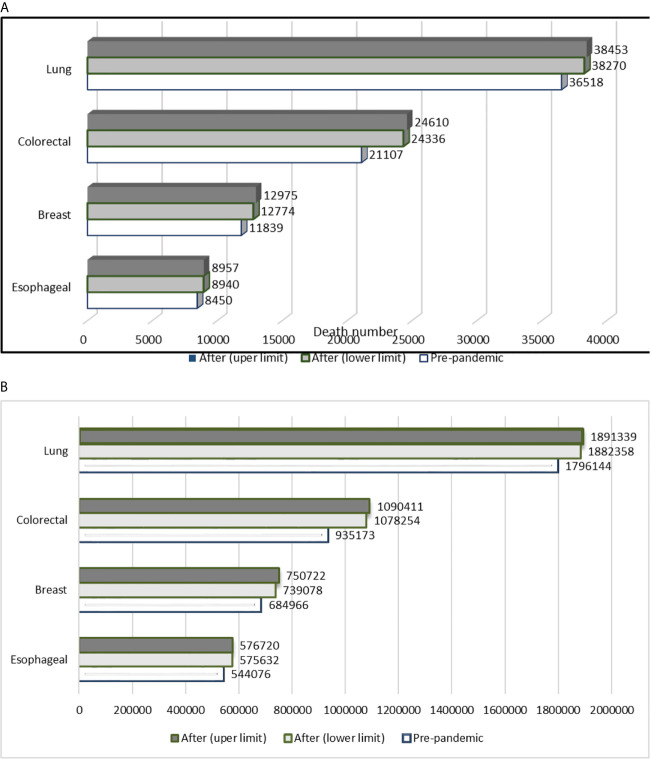
**(A)** Estimated cumulative number of deaths in the UK due to breast, colorectal, lung, and esophageal cancers up to year 5 after the diagnosis. **(B)** Estimated cumulative numbers of deaths throughout the world due to breast, colorectal, lung, and esophageal cancer up to 5 years after the diagnosis (Calculated numbers are an estimate). Based on the data published by Maringe and coworkers, we anticipate a minimum 7.9–9.6%, 15.3–16.6%, 4.8–5.3%, and 5.8–6.0% increase in the numbers of deaths due to breast, colorectal, lung, and esophageal cancer, respectively, up to year 5 after the diagnosis (38, 39).

The estimated cumulative numbers of cancer deaths in the UK up to year 5 after the diagnosis are shown in **Figure 3A**. This figure was extrapolated from the total number of cancer deaths in the UK (total breast cancer deaths n=11,839; colorectal cancer deaths n=21,107, lung cancer deaths n=36,518; and esophageal cancer deaths n=8450) reported by the International Agency for Research on Cancer (IARC) (39). Maringe et al. anticipate 7.9–9.6%, 15.3–16.6%, 4.8–5.3%, and 5.8–6.0% increases in the numbers of deaths due to breast, colorectal, lung, and esophageal cancers, respectively, up to year 5 after the diagnosis (38).

Cancer deaths due to COVID-19 throughout the world were calculated on the basis of the cancer database IARC (39) and a published study (38). Delayed cancer screening is estimated to cause the following additional numbers of cancer deaths secondary to breast, esophageal, lung, and colorectal cancer, respectively: 54,112–65,756, 31,556–32,644, 86,214–95,195, and 143,081–155,238 in the worldwide (**Figure 3B**).

Any interruption in cancer services without adequate strategies to accommodate individuals who missed their screening would lead to years of life lost. Maringe and co-workers determined the numbers of years of life lost due to additional deaths attributable to four cancer types (breast, lung, colorectal, and esophageal) at five years, and reported an anticipated 59,204 to 63,229 YLLs secondary to additional deaths (3291 to 3621) from the four cancer types during the first five years after diagnosis (38). Yong et al. estimated that a six-month interruption in breast and colorectal cancer screening would potentially result in 8000 (95% CI 3500–12,000) years of life lost over the lifetime (17). These statistics clearly underline the fragility of established health care systems. Indeed, the COVID-19 pandemic could challenge the resilience of health programs in oncology. The major interruption of cancer services may well be expected to neutralize achievements in the field for a whole decade. The estimated effects of COVID-19 on mortality rates and YLLs are summarized in **Table 4**.

**Table 4:** Impact of COVID-19 on mortality rates and YLLs in cancer patients.

First Author	Country	Study Design/source of the data	Quality Assessment	Cancer type or screening (sample size)	Additional death	Decline in the survival of cancer patients at 5 years after diagnosis:
**Maringe** (38)	England	National population-based Modelling study (National Cancer Registration)		Total number of cancer patients (93,607)	Additional deaths due to these four cancer types at 5 years after diagnosis: 3291 and 3621.	
			Good	Breast (32,583)	281 (95% CI 266–295) to 344 (329–358) additional deaths; a 7.9–9.6% increase	1%
				Lung (29,305)	1235 (1220–1254) to 1372 (1343–1401) additional deaths, a 4.8–5.3% increase	3.5%
				CRC (24,975)	1445 (1392–1591) to 1563 (1534–1592) additional deaths, a 15.3–16.6% increase	6.4%
				Esophageal (6,744)	330 (324–335) to 342 (336–348) additional deaths, a 5.8–6.0% increase up to 5 years after diagnosis.	6.1–6.3%
**Yong** (17)	Canada	Simulation modeling analysis (Canadian Cancer Registry)	Good	Breast	A three-, six-, and 12- month service interruption with immediate restoration of screening would lead to 110, 250, and 480 cumulative excess breast cancer deaths, respectively, between 2020 and 2029. If a 24-month transition period of reduced breast screening volumes followed the interruption, the number of deaths for a six- and 12-month interruption would increase from 250 to 730 and 480 to 930 deaths, respectively.	
				CRC	A six- and 12- month service interruption with immediate restoration of screening would lead to 450 and 930 cumulative excess colorectal cancer deaths, respectively, between 2020 and 2029. If a 24-month transition period of reduced colorectal screening volumes followed the interruption, the number of deaths for a six- and 12-month interruption would increase from 450 to 1150 and 930 to 1800 deaths, respectively.	

YLLs, Years of life lost; n, Number; CRC, Colorectal cancer.

## Discussion

COVID-19 is believed to have influenced cancer screening programs (7). The present systematic review was designed to investigate the impact of COVID-19 on cancer screening and suggest global measures to counteract future threats. The effect of the COVID-19 pandemic on cancer screening and diagnosis is expected to result in more numerous advanced cancers as well as increase cancer mortality rates and YLLs.

In the present study, all published studies by countries with different prevalence of COVID-19 have been reviewed. Based on the report WHO, COVID-19 in the United States, England, and Italy has been highly prevalent, whereas Taiwan had a lowly prevalence (40). Published studies have disclosed a marked decline in cancer screening, diagnostic imaging, as well as histopathological and cytological biopsies during the COVID-19 pandemic (8, 14, 26, 27, 30, 31, 34, 36, 37, 41). The reduction has been attributed to stay-at-home orders, patients’ fear of infection, hesitation to seek care, the perceived risk of exposure to COVID-19 for clinicians, changing hospital policies in re-deployment of staff towards critical care for the management of COVID-19 patients, triage of patients with COVID-19 infection, and the cessation of cancer screening in hospitals (28).

Short-term (3-6 months) and long-term (>12 months) interruption of cancer screening will delay the diagnosis of cancers and cause a shift in favor of more advanced cancers (15, 17, 31, 36, 38, 42). Cancer in advanced stages requires more complex care, is associated with a lower likelihood of response to therapy and cure of the disease, higher costs (10, 15, 28, 43), and graver outcomes (8). Despite limited evidence, Yin et al. showed that a short-term interruption (3-4 months) of cancer screening may not necessarily influence cancer stage. Evidently, the subject calls for further investigation (16).

In a national, population-based modeling study in England, Maringe et al. reported that delays in cancer diagnosis would lead to excess short-term cancer mortality and 40,000 years of life lost (38).

On account of delays in cancer diagnosis in Canada, Yong et al. predicted approximately 5300 additional breast cancer deaths and 4500 additional deaths due to colorectal cancer (17). Sud et al. claimed that in England a delay of 3–6 months in cancer screening and surgery will exert a significant impact on survival and mitigate 19–43% of life-years gained (44).

The suspension of cancer screening or cancer prevention programs may be expected to aggravate the patients’ suffering, disease burden, mortality rates at 5 years, the economic burden, and the workload for surgeons and oncologists (36, 38). Even after the resumption of routine diagnostic services, the current backlogs in medical and surgical subspecialties will cause substantial delays (38). On the other hand, a surge in diagnosed cancer diagnosis is expected when screening resumes (17).

Current projections indicate that the COVID-19-related disruption may last for 18 months or longer. The medical community and the world at large will be faced with significant challenges in the near future. Cancer screening programs must be implemented despite the lack of information for health care workers and patients concerning their risk of contracting COVID-19 from various health care interactions. Especially in the current setting, we need policies to promote access to cancer care, support the cancer care ecosystem, and minimize morbidity and mortality rates during and after the pandemic.

- Measures should be instituted at the health system level (national or jurisdictional) as well as the health service level (including specialist and primary care, in both the public and private sectors) to identify and address system-wide cancer control as well as support high-quality cancer care.
- Cancer screening services should be resumed early while respecting infection prevention (social distancing, using personal protective equipment [PPE], vaccination etc.). Prioritization of health care and reallocation of resources will be needed to minimize the negative impact of delayed diagnosis and therapy for oncological patients. Simultaneously, adequate numbers of health care workers should be assigned and suitable spaces provided for screening in order to ensure timely care for patients with alarm symptoms. A proportionate increase in funds earmarked for health care should also be taken into account.
- Raising diagnostic resources is complex because it requires effective coordination across all hospital subspecialties and not just in specialized cancer teams.
- Dividing institutions into Covid-dedicated hospitals and Covid-free institutions may be useful to reduce the risk and fear of infection among non-COVID patients. Hospital staff would require less protective equipment at Covid-free hospitals. The latter would also not experience any shortage of personnel for performing elective high-risk procedures.
- After a brief period of training, less specialized staff such as general practitioners (GP), midwives or nurses could be involved in screening programs. National cancer societies should establish guidelines and design a screening questionnaire for risk stratification. The screening questionnaires could include specific questions such as the number of first-degree relatives with cancer, whether a screening investigation (colonoscopy, mammography, etc.) was performed for non-screening purposes over the last 5–10 years, and with what results.
- Outreach services could be provided to enhance access to health services and improve overall retention at the national level. Outreach services would ensure the stability of cancer screening in a similar public health crisis.
- Policy-makers would be well advised to consider the establishment of a flexible outreach system into the community as a preparation for future pandemics.
- Public health messaging should accurately convey the risk of severe illness due to COVID-19 versus the risk of not seeking healthcare advice if patients are symptomatic, and provide evidence-based information for clinicians to balance the risks for patients against the benefits of medical procedures during the pandemic.
- Cancer screening education apps *via* smartphones or tablet devices should be used to educate the population about cancer screening.
- Dedicated cancer awareness programs will need to consider a wide range of media channels in order to reach their target groups. A media-led education intervention could be used to enhance the awareness of the public and their attendance of screening tests.
- Cancer programs should encompass telemedicine and teleworking to ensure the continuum of care without losing clinical and human contact. Technology is increasingly used across the health care system to improve the quality and efficiency of care. Artificial intelligence (AI) has the potential to transform the delivery of cancer care. The pandemic warrants further development of artificial intelligence in medical research and its use in optimizing cancer care (45).
- Further measures include self-sampling, such as that employed for HPV testing, the use of rapid diagnostic kits (Fecal Immunochemical Test or FIT) and the return the samples by postal mail. Self-sampling is believed to be equivalent to clinician sampling and can be taught by simple graphics or animated video presentation.

The world is currently in a state of shock. Urgent policy interventions are needed to handle the backlog of routine diagnostic services and minimize the effect of the COVID-19 pandemic on cancer patients. While the majority of published studies have been focused on the short-term (three- or six-month) effects of interrupted cancer screening, some countries had been involved with COVID-19 for more than a year. According to the WHO prediction, the second year of COVID-19 will be more difficult because of the emergence of new variants. Further negligence of cancer screening programs will intensify the crisis. Experts around the world should work together to develop a protocol that will minimize the consequences of this problem. When we return to our lifestyles prior to the COVID-19 crisis, we will be faced with the following question: to what extent have survival standards and our overall quality of life been affected by the change in cancer screening programs? Investigators all over the world will be faced with the task of collecting data in order to answer this critical question.

In summary, changes in cancer services due to international pandemic measures are expected to result in a large number of additional cancer deaths. A significant decline in cancer screening and biopsy sampling is expected that in the short term reduce cancer diagnosis rates and in the long term increase cancer diagnosis rates, advanced cancers, mortality rates and years of life lost (YLLs). The reason for these long-term sequelae can be traced to the unpreparedness of the world for such a catastrophe. Indeed, in the initial phases of the pandemic, it was estimated that the problem would be resolved in a few months and all delays would be compensated. However, the sheer duration of the pandemic affected health policies throughout the world.

## Limitations

The use of English-language articles might have limited the results of this study. Crucial data published in other languages may have been omitted. The small sample size of some studies and the use of easy and accessible sampling may have impaired the generalizability of the studies. The strengths of the study may be summarized as follows: it is a comprehensive review of the impact of COVID-19 on all cancer screening programs, and provides practical suggestions for dealing with COVID-19 during and after the current pandemic.

## Author Contributions

All authors contributed in all sections and writing. All authors contributed to the article and approved the submitted version.

## Conflict of Interest

The authors declare that the research was conducted in the absence of any commercial or financial relationships that could be construed as a potential conflict of interest.
